# RETRACTION: A Meta‐Analysis of the Effect of Laparoscopic Gastric Resection on the Surgical Site Wound Infection in Patients With Advanced Gastric Cancer

**DOI:** 10.1111/iwj.70315

**Published:** 2025-03-06

## Abstract

**RETRACTION:** HanX. , KongD. , HeX. , XieS. , and LiC. , “A Meta‐Analysis of the Effect of Laparoscopic Gastric Resection on the Surgical Site Wound Infection in Patients With Advanced Gastric Cancer,” International Wound Journal 20, no. 10 (2023): 4300–4307, 10.1111/iwj.14332.37493021 PMC10681515

The above article, published online on 26 July 2023, in Wiley Online Library (http://onlinelibrary.wiley.com/), has been retracted by agreement between the journal Editor in Chief, Professor Keith Harding; and John Wiley & Sons Ltd. Following an investigation by the publisher, all parties have concluded that this article was accepted solely on the basis of a compromised peer review process. The editors have therefore decided to retract the article. The authors did not disagree with the retraction.

**RETRACTION:** X. Han , D. Kong , X. He , S. Xie , and C. Li , “A Meta‐Analysis of the Effect of Laparoscopic Gastric Resection on the Surgical Site Wound Infection in Patients With Advanced Gastric Cancer,” International Wound Journal 20, no. 10 (2023): 4300–4307, 10.1111/iwj.14332.37493021 PMC10681515

The above article, published online on 26 July 2023, in Wiley Online Library (http://onlinelibrary.wiley.com/), has been retracted by agreement between the journal Editor in Chief, Professor Keith Harding; and John Wiley & Sons Ltd. Following an investigation by the publisher, all parties have concluded that this article was accepted solely on the basis of a compromised peer review process. The editors have therefore decided to retract the article. The authors did not disagree with the retraction.

